# Analysis of factors influencing the burden on family caregivers of disabled older adults in Guangzhou

**DOI:** 10.3389/fpubh.2025.1628022

**Published:** 2025-08-21

**Authors:** Danrong Lin, Junbin Liao, Yanxi Liu, Yuquan Zhong, Jingyun Pan, Heng Fang, Yunfeng Lin, Yilu Yao, Jiewen Su, Bingjie Hu, Wei Zhu

**Affiliations:** ^1^Institute of Public Health, Guangzhou Center for Disease Control and Prevention, Guangzhou Medical University, Guangzhou, China; ^2^Department of Scientific Research, Guangzhou Center for Disease Control and Prevention, Guangzhou, China; ^3^Shenzhen Centre for Disease Control and Prevention, Shenzhen, China; ^4^Philosophy and Social Sciences Key Laboratory of Guangdong Higher Education Institutes for Health Governance Based on Big Data Utilization, Guangzhou, China

**Keywords:** old adult, disability, family caregivers, caregiving burden, influencing factors

## Abstract

**Objective:**

This study aims to investigate the current status of disability among older adults and analyze factors influencing the burden on their family caregivers in southern China.

**Methods:**

A cross-sectional survey was conducted among 334 pairs of disabled older adults (≥65 years) and their primary family caregivers across three districts in Guangzhou. Face-to-face interviews using standardized questionnaires assessed disability levels via the “Long-term Care Disability Level Assessment” scale and caregiver burden via the Zarit Burden Interview (ZBI) scale. For ordinal variables, the Kruskal-Wallis H test was applied for multi-group comparisons, while the Mann–Whitney U test was used for two-group analyses. Statistical significance was set at *p* < 0.05. Ordinal logistic regression identified factors associated with caregiver burden.

**Results:**

Among 334 disabled older adults in Guangzhou, the mean age was 82 years. Of these, 55.7% were married, 43.4% were male, 43.4% had an educational attainment of primary school or below. For the 334 family caregivers, 62.3% were female, 36.8% had completed high school or vocational education. The median years of caregiving experience was 5 years, with a median weekly caregiving time of 70 h. Caregiver burden distribution was as follows: 16.2% no burden, 41.0% mild burden, 28.7% moderate burden, and 14.1% severe burden. Statistically significant differences in caregiver burden were observed across the following variables (*p* < 0.05): disabled old adults’ activities of daily living (ADL), caregivers’ gender, child in school, physical disease, years of caregiving experience, weekly caregiving time, social interaction frequency, life satisfaction, and social support network size. Higher caregiver life satisfaction and elevated ADL scores in care recipients emerged as protective factors. Conversely, longer weekly caregiving hours and caregivers’ physical illness were identified as risk factors.

**Conclusion:**

This study identifies weekly caregiving time, caregivers’ physical disease, disabled older adults’ ADL capacity, and caregivers’ life satisfaction as critical determinants of burden severity in aging China, advocating multilevel interventions.

## 1 Introduction

In 2011, the World Health Organization (WHO) defined disability as an umbrella term encompassing impairments, activity limitations, and participation restrictions (1). According to the Seventh National Population Census, individuals aged 60 and above account for 18.7% of China’s total population, reflecting a 5.44% increase compared to the Sixth National Population Census. Within this aging population, a significant proportion of older adults experience disability due to aging, chronic diseases, or other factors.

In traditional Chinese culture, filial piety encourages older adults to age at home rather than in institutional care settings. Research based on the China Health and Retirement Longitudinal Study dataset indicates that family-based care remains predominant in China (2). A research shows that 5.9% of disabled old adults in rural China report receiving formal care, while the proportion in urban areas is 36.9% (3). Disability restricts mobility, narrows social interactions, and exacerbates psychological issues such as anxiety and depression among older adults (4). However, disability not only severely impacts the quality of life of older adults but also imposes substantial burdens on their family caregivers. In 1986, Zarit et al. (5) defined caregiver burden as the perceived deterioration of caregivers’ emotional or physical health, social life, and financial status resulting from providing care to relatives.

A review on caregiver support highlights that caregiving adversely affects caregivers’ physical and mental health, exposing them to risks such as reduced quality of life and heightened isolation (6). Furthermore, disabled older adults often require long-term medical and daily care, leading to high healthcare costs, rehabilitation expenses, and family caregiving expenditures, which significantly strain household finances. Prolonged caregiving may also trigger familial conflicts and strain interpersonal relationships. At the societal level, the growing aging population and rising prevalence of disability among older adults intensify pressure on healthcare systems. Long-term care, rehabilitation, and management of disability-related complications demand substantial medical resources, increasing service demands and exacerbating resource allocation challenges. Direct and indirect economic costs associated with disability—including household and national medical expenditures, long-term care costs, and investments in aging-related infrastructure—amplify the burden on social security systems. Andrew Kingston et al. (7) found that increased survival years among older populations impose significant strains on public health systems.

As the capital of Guangdong Province, Guangzhou is a core metropolis in this populous region, hosting a large aging population. The Seventh National Population Census reports that 2,130,600 individuals in Guangzhou (11.41% of its population) are aged 60 or older, marking a 1.67 percentage-point increase compared to the 2010 census. The old urban areas of Guangzhou have entered a phase of deep aging. With accelerating population aging, the number of disabled older adults continues to rise annually. Comorbidities such as hypertension and diabetes among disabled older adults further escalate medical demands, posing significant challenges to the city’s healthcare system and necessitating more equitable resource allocation. By the end of 2022, Guangzhou had 195,700 healthcare professionals (a 4.26% increase) and 110,500 hospital beds (a 3.75% increase). Despite these improvements in healthcare resources, persistent issues such as uneven resource distribution, shortages of specialized care professionals, and gaps in long-term care insurance systems hinder Guangzhou’s capacity to fully address the growing disabled population. As a major economically developed and densely populated city in China, Guangzhou’s challenges in managing the burden on disabled older adults and their family caregivers exemplify the broader societal struggles posed by aging populations.

## 2 Method

### 2.1 Sample size calculating

The formula for sample size calculation is: $n=\frac{Z_{\frac{\alpha }{2}}^{2}\;P(1-P)}{d^{2}}\times \text{deff}$ . *p* = 20.8% (8), α = 0.05, d = 0.05, Deff = 1.3. The sample size was calculated to be 320, meaning that at least 320 pairs of disabled old adults and their family caregivers need to participate in the study.

### 2.2 Participant recruitment

Guangzhou’s acknowledged regional development hierarchy comprises established downtown, new urban districts, inner suburbs and outer suburbs. Considering sample representativeness and research resources (time, funding, personnel), we randomly selected one district each from established downtown, new urban districts, and inner suburbs. From each district, 100–150 disabled older adults and their family caregivers were randomly selected for surveying. 343 pairs of disabled old adults and their family caregivers were invited to participate in the study. 9 pairs subsequently withdrew from the study. The final analytical sample thus comprised 334 pairs who completed the full study protocol. Through collaboration with community health service centers in the sampled areas, initial telephone contact was made with households containing adults aged ≥65 years. After explaining the study’s purpose and significance, preliminary eligibility screening was conducted with both older adults and their caregivers. Those meeting all inclusion/exclusion criteria were formally invited to participate in this study. Data collection involved two-person interviewer teams conducting in-home, face-to-face interviews using paper-based questionnaires.

The inclusion criteria for disabled old adults were as follows: aged 65 years or older and assessed as disabled. The exclusion criteria included congenital disability or disability resulting from accidental injuries. For participants who met any of the following conditions, part of the questionnaire was completed by a family caregiver familiar with their situation: those with acute illnesses or chronic diseases in acute exacerbation; those with severe or terminal illnesses; those with severe cognitive impairments (e.g., Alzheimer’s disease, mental illnesses); and those with hearing impairments or other conditions that prevented effective communication.

The inclusion criteria for family caregivers were as follows: being a family member and the primary caregiver of the disabled old adults. The exclusion criteria included inability to complete the survey or unwillingness to cooperate fully during the survey process.

This study was reviewed and approved by the Ethics Committee of the Guangzhou Center for Disease Control and Prevention (Approval Number: GZCDC-ECHR-2023P0081). Informed consent was obtained from all participants.

### 2.3 Assessment of disability

This study employed the Long-term Care Disability Level Assessment standard issued by the China Healthcare Security Administration to comprehensively evaluate the disability status and severity of older adults individuals across three domains: activities of daily living (ADL), cognitive ability, and sensory and communication abilities.

Activities of Daily Living (ADL): The ADL assessment evaluates 10 daily activities: facial and oral hygiene, bathing, eating, dressing, bowel control, bladder control, toileting, stair climbing, walking on flat ground, and transferring between bed and chair. Based on the total score, the ability to perform daily activities is categorized into three levels: severe impairment (0–40 points), moderate impairment (45–60 points), and mild impairment (65–95 points).

Cognitive Ability: Cognitive function was evaluated across four dimensions: time orientation, spatial orientation, person orientation, and memory. Each dimension was scored on a 0–4 scale, with lower scores reflecting poorer performance. Total scores classified cognitive ability into four levels: severe impairment (0–1 points), moderate impairment (2–3 points), mild impairment (4–15 points), and no impairment (16 points).

Sensory and Communication Abilities: This domain assessed vision, hearing, and communication ability, with each aspect scored on a 0–4 scale (lower scores indicating poorer ability). Total scores categorized sensory and communication abilities into four levels: severe impairment (0–1 points), moderate impairment (2–3 points), mild impairment (4–11 points), and no impairment (12 points).

The Long-term Care Disability Level Assessment categorizes disability into six grades based on scores in activities of daily living (ADL), cognitive ability, and sensory and communication abilities: mild, moderate, and severe (with severe disability further divided into Grades 1, 2, and 3). In this study, disability severity was consolidated into three levels: mild, moderate, and severe, with all severe subgrades (Grades 1–3) classified uniformly as severe disability.

### 2.4 Assessment of caregiver burden

Caregiver burden was evaluated using the Zarit Burden Interview (ZBI) scale (9), a psychometric instrument developed by Zarit et al. in the early 1980s to assess the multidimensional burden experienced by long-term caregivers of individuals with chronic illnesses. The ZBI quantifies physiological, psychological, social, and economic burdens arising from caregiving responsibilities.

This scale comprehensively addresses critical aspects of caregiver burden, including physical and mental health, social engagement, and financial strain. Each item is rated on a 5-point Likert scale: Never (0), Rarely (1), Sometimes (2), Often (3), and Always (4). Caregivers self-report their experiences based on subjective perceptions, with higher scores indicating greater burden severity. Total scores range from 0 to 88, categorized as follows: No burden(0–19points), Mild burden (20–39points), Moderate burden(40–59points), Severe burden (60–88points).

### 2.5 Survey content

This study collected the following characteristics of disabled old adults: age, gender, marital status, educational attainment, monthly household income per capita, the number of diseases, activities of daily living (ADL), cognitive ability, sensory and communication abilities, long-term care insurance, disability certificate. For family caregivers, variables included age, gender, marital status, educational attainment, occupation, child in school, relationship to the disabled old adults, physical diseases, hired nurse, acquisition of caregiving knowledge/skills, years of caregiving experience, weekly caregiving time, social interaction frequency, life satisfaction, and social support network size.

#### 2.5.1 Variable classifications

Gender: Male or female.

Marital status: Married, widowed, or others.

Educational attainment: Primary school or below, junior high school, senior high school/technical secondary school, or college degree and above.

Monthly household income per capita: <¥1,000; ¥1,000–2,999; ¥3,000–4,999; ¥5,000–9,999; ≥¥10,000.

The number of diseases: 0 ~ 2, 3 ~ 6, ≥7.

Activities of daily living (ADL): mild impairment, moderate impairment, severe impairment.

Cognitive ability: no impairment, mild impairment, moderate impairment, severe impairment.

Sensory and communication abilities: no impairment, mild impairment, moderate impairment, severe impairment.

Long-term care insurance: yes, no.

Disability certificate: yes, no.

Occupation: Retired, laborer/farmer/part-time worker, unemployed, or others.

Child in school: yes, no.

Relationship to care recipient: Spouse, child/grandchild, or others.

Physical disease: yes, no. (Family caregivers experiencing physical disease in this study reported no limitations in their daily activities due to their disease.)

Hired nurse: yes, no.

Acquisition of caregiving knowledge/skills: yes, no.

Years of caregiving experience: 0–5 years; >5 years.

Weekly caregiving time: 0–30 h; 31–60 h; >60 h.

Social interaction frequency: Daily or 1–6 times weekly; 1–3 times monthly; 1–11 times annually; never.

Life satisfaction: Very satisfied, mostly satisfied, neutral, dissatisfied, or very dissatisfied.

Social support network size: 0 individuals; 1–2 individuals; 3–5 individuals; ≥6 individuals.

### 2.6 Data analysis

This study utilized the EPiData software for parallel double-entry data management, converting paper-based questionnaires into electronic formats. Discrepancies between the two data entries were systematically compared and rectified through verification to ensure data accuracy and completeness. Personal identifiers (e.g., names and ID numbers) were replaced with anonymous codes during data entry to uphold privacy protection and confidentiality principles.

The dataset underwent rigorous logical consistency checks to identify contradictions or implausible values. Missing data were addressed via multiple imputation or median substitution, with explicit documentation of imputation status. Outliers were identified and processed by consulting field investigators to verify authenticity, followed by corrections to maintain data validity and reliability.

Categorical variables were summarized as frequencies and percentages. For ordinal variables, the Kruskal-Wallis H test was applied for multi-group comparisons, while the Mann–Whitney U test was used for two-group analyses. Statistical significance was set at *p* < 0.05. Multivariable ordered logistic regression was employed to assess associations. All statistical analyses were performed using IBM SPSS Statistics 26.

### 2.7 Quality control protocol

Questionnaire Design Phase: Scientific content validity was ensured through unambiguous items with exhaustive response options; iterative refinement occurred post-pilot testing.

Sampling Phase: Randomized sampling procedures were strictly implemented, with rigorous application of inclusion/exclusion criteria.

Training Phase: Surveyors responsible for scheduling mastered: (1) appointment protocols, (2) functional disability assessment methods. Field interviewers demonstrated competency in: (1) communication techniques, (2) standardized assessment, (3) instrument administration, (4) sensitive issue handling, and (5) professional etiquette. Certification through competency assessment was mandatory prior to fieldwork. Burden assessments required caregiver evaluation in the absence of care recipients to minimize response bias.

Field Implementation: Dyadic interviewer teams conducted home visits, performing real-time cross-verification to ensure data fidelity.

Data Management: (1) Daily completeness audits with immediate remediation of missing data; (2) Double-blind electronic data entry with consistency reconciliation; (3) Logic checks, outlier handling, and error correction protocols.

## 3 Results

Among the 334 disabled old adults, the mean age was 82 years. Disability severity was distributed as follows: mild disability (106 cases, 31.7%), moderate disability (67 cases, 20.6%), and severe disability (161 cases, 48.2%). Among their family caregivers, the mean age was 64 years, with caregiver burden levels categorized as no burden (54 cases, 16.2%), mild burden (137 cases, 41.0%), moderate burden (96 cases, 28.7%), and severe burden (47 cases, 14.1%).

As shown in Table 1, caregiver burden significantly differed across activities of daily living (ADL) levels among disabled older adults individuals (*p* < 0.05). Similarly, Table 2 demonstrates statistically significant variations in caregiver burden among family caregivers based on gender, years of caregiving experience, weekly caregiving hours, physical disease, social interaction frequency, life satisfaction, and social support network size (all *p* < 0.05).

**Table 1:** Comparison of caregiver burden of disabled older adults with different characteristics in Guangzhou.

Variables	Classification	Number of Participants	No Burden (n = 54) %		Mild Burden (n = 137) %		Moderate Burden (n = 96) %		Severe Burden (n = 47) %		*Z/H*	*p*值
Gender	Male	145	17	11.7	58	40.0	49	33.8	21	14.5	−1.556	0.120
	Female	189	37	19.6	79	41.8	47	24.9	26	13.8		
Age	65 ~ 80 years	154	22	14.3	57	37.0	46	29.9	24	15.6	−1.255	0.210
	≥81 years	180	32	17.8	80	44.4	50	27.8	23	12.8		
Marital status	Married	186	28	15.1	75	40.3	57	30.6	26	14.0	0.248	0.884
	Widowed	136	25	18.4	55	40.4	36	26.5	20	14.7		
	Others	12	1	8.3	7	58.3	3	25.0	1	8.3		
Educational attainment	Primary school or below	145	32	22.1	56	38.6	37	25.5	20	13.8	3.368	0.643
	Junior high school	80	6	7.5	38	47.5	27	33.8	9	11.3		
	Senior high school/technical secondary school	69	12	17.4	26	37.7	16	23.2	15	21.7		
	College degree and above	40	4	10.0	17	42.5	16	40.0	3	7.5		
Monthly household income per capita	<¥1,000	16	2	12.5	9	56.3	4	25.0	1	6.3	1.180	0.881
	¥1,000–2,999	62	12	19.4	22	35.5	15	24.2	13	21.0		
	¥3,000–4,999	153	23	15.0	66	43.1	44	28.8	20	13.1		
	¥5,000–9,999	82	14	17.1	28	34.1	27	32.9	13	15.9		
	≥¥10,000	21	3	14.3	12	57.1	6	28.6	0	0.0		
The number of diseases	0 ~ 2	57	10	17.5	24	42.1	18	31.6	5	8.8	1.484	0.476
	3 ~ 6	245	39	15.9	105	42.9	65	26.5	36	14.7		
	≥7	32	5	15.6	8	25.0	13	40.6	6	18.8		
Long-term care insurance	Yes	129	39	19.0	80	39.0	55	26.8	31	15.1	−1.463	0.143
	No	205	14	15.6	40	44.4	26	28.9	10	11.1		
Disability Certificate	Yes	90	40	16.4	97	39.8	70	28.7	37	15.2	−0.086	0.932
	No	244	25	15.9	48	30.6	22	14.0	12	7.6		
Activities of Daily Living	Mild impairment	157	9	14.1	26	40.6	25	39.1	7	10.9	12.473	0.002
	Moderate impairment	64	20	12.5	63	39.4	49	30.6	28	17.5		
	Severe impairment	160	7	24.1	12	41.4	3	10.3	7	24.1		
Cognitive Ability	No impairment	29	39	15.2	102	39.7	84	32.7	32	12.5	2.901	0.407
	Mild impairment	257	5	19.2	13	50.0	3	11.5	5	19.2		
	Moderate impairment	26	3	13.6	10	45.5	6	27.3	3	13.6		
	Severe impairment	22	8	25.8	11	35.5	8	25.8	4	12.9		
Sensory and Communication Abilities	No impairment	31	44	15.2	120	41.4	86	29.7	40	13.8	4.325	0.228
	Mild impairment	290	2	20.0	5	50.0	2	20.0	1	10.0		
	Moderate impairment	10	0	0.0	1	33.3	0	0.0	2	66.7		
	Severe impairment	3	17	11.7	58	40.0	49	33.8	21	14.5		

**Table 2:** Comparison of caregiver burden of family caregivers with different characteristics in Guangzhou.

Variables	Classification	Number of Participants	No Burden (n = 54) %		Mild Burden (n = 137) %		Moderate Burden (n = 96) %		Severe Burden (n = 47) %		*Z/H*	*p*值
Gender	Male	126	29	23.0	50	39.7	31	24.6	16	12.7	−2.625	0.009
	Female	208	25	12.0	87	41.8	65	31.3	31	14.9		
Age	0–50 years	184	31	16.8	82	44.6	44	23.9	27	14.7	9.806	0.347
	>50 years	150	23	15.3	55	36.7	52	34.7	20	13.3		
Marital status	Married	297	48	16.2	126	42.4	85	28.6	38	12.8	−0.739	0.460
	Others	37	6	16.2	11	29.7	11	29.7	9	24.3		
Educational attainment	Primary school or below	42	8	19.0	19	45.2	12	28.6	3	7.1	1.376	0.711
	Junior high school	80	11	13.8	34	42.5	22	27.5	13	16.3		
	Senior high school/technical secondary school	123	23	18.7	44	35.8	40	32.5	16	13.0		
	College degree and above	89	12	13.5	40	44.9	22	24.7	15	16.9		
Occupation	Retired	246	35	14.2	100	40.7	78	31.7	33	13.4	4.959	0.175
	Laborer/farmer/part-time worker	18	2	11.1	7	38.9	7	38.9	2	11.1		
	Unemployed	39	7	17.9	17	43.6	3	7.7	4	10.3		
	Others	31	10	32.3	13	41.9	8	25.8	8	25.8		
Child in school	Yes	49	42	85.7	116	236.7	84	171.4	43	87.8	−2.121	0.034
	No	285	12	4.2	21	7.4	12	4.2	4	1.4		
Hired nurse	Yes	54	7	13.0	26	48.1	13	24.1	8	14.8	−0.321	0.748
	No	280	47	16.8	111	39.6	83	29.6	39	13.9		
Learn caregiving knowledge and skills	Yes	171	32	18.7	65	38.0	49	28.7	17	9.9	−0.078	0.938
	No	163	22	13.5	72	44.2	47	28.8	30	18.4		
Years of caregiving experience	0–5 years	179	35	19.6	78	43.6	52	29.1	14	7.8	−3.111	0.002
	>5 years	155	19	12.3	59.0	38.1	44	28.4	33	21.3		
Weekly caregiving time	0–30 h	70	18	25.7	36	51.4	11	15.7	5	7.1	18.932	<0.001
	31–60 h	79	11	13.9	35	44.3	22	27.8	11	13.9		
	>60 h	185	25	13.5	66	35.7	63	34.1	31	16.8		
Physical disease	Yes	219	27	12.3	94	42.9	63	28.8	35	16.0	−2.184	0.029
	No	115	27	23.5	43	37.4	33	28.7	12	10.4		
Social interaction frequency	Daily or 1–6 times weekly	90	20	22.2	41	45.6	21	23.3	8	8.9	9.528	0.023
	1–3 times monthly	122	19	15.6	43	35.2	38	31.1	22	18.0		
	1–11 times annually	101	12	11.9	46	45.5	29	28.7	14	13.9		
	Never	21	3	14.3	7	33.3	8	38.1	3	14.3		
Life satisfaction	Very satisfied	25	10	40.0	10	40.0	3	12.0	2	8.0	42.376	<0.001
	Mostly satisfied	140	24	17.1	68	48.6	40	28.6	8	5.7		
	Neutral	105	17	16.2	39	37.1	36	34.3	13	12.4		
	Dissatisfied	46	2	4.3	16	34.8	12	26.1	16	34.8		
	Very dissatisfied	18	1	5.6	4	22.2	5	27.8	8	44.4		
Social support network size	0 individuals	153	18	11.8	62	40.5	48	31.4	25	16.3	12.734	0.005
	1–2 individuals	89	11	12.4	19	21.3	11	12.4	4	4.5		
	3–5 individuals	45	17	37.8	41	91.1	20	44.4	11	24.4		
	≥6 individuals.	47	8	17.0	15	31.9	17	36.2	7	14.9		

An ordinal logistic regression analysis was conducted with caregiver burden in Guangzhou City as the dependent variable (0 = no burden, 1 = mild burden, 2 = moderate burden, 3 = severe burden) and nine independent variables: activities of daily living (ADL) of disabled old adults, caregivers’ gender, presence of school-aged children, caregiving duration (years), daily care hours, physical health conditions, social engagement frequency, life satisfaction, and social support. The parallel lines test demonstrated model adequacy (χ^2^ = 35.782, *p* = 0.572). As shown in Table 3, better ADL(OR = 0.992, 95% CI = 0.984–0.999) in old adults and caregivers’ better life satisfaction(very satisfied: OR = 0.063, 95% CI = 0.018–0.213; mostly satisfied: OR = 0.179, 95% CI = 0.068–0.0469; neutral: OR = 0.266, 95% CI = 0.102–0.695) emerged as protective factors against caregiver burden. Conversely, longer daily caregiving time(OR = 1.009, 95% CI = 1.002–1.015) and caregivers’ physical illnesses(OR = 1.642, 95% CI = 1.057–2.552) were identified as significant risk factors for elevated burden levels.

**Table 3:** An ordered logistic regression analysis of factors influencing the burden on family caregivers of disabled older adults individuals in Guangzhou.

Variables	Classification	β	$S_{\bar{x}}$	Wald *x^2^*值	*p*值	*OR*值	95%*CI*
Caregivers’ gender	Male	−0.315	0.222	2.016	0.156	0.730	0.472 ~ 1.127
	Female	0	.	.	.	1.000	
Child in school	Yes	0.296	0.308	0.921	0.337	1.344	0.735 ~ 2.457
	No	0	.	.	.	1.000	
Caregivers’ physical disease	Yes	0.496	0.225	4.86	0.027	1.642	1.057 ~ 2.552
	No	0	.	.	.	1.000	
Social interaction frequency	Daily or 1–6 times weekly	−0.466	0.464	1.008	0.315	0.628	0.253 ~ 1.559
	1–3 times monthly	−0.051	0.45	0.013	0.91	0.950	0.393 ~ 2.296
	1–11 times annually	−0.381	0.456	0.698	0.403	0.683	0.28 ~ 1.669
	Never	0	.	.	.	1.000	
Life satisfaction	Very satisfied	−2.768	0.624	19.655	0	0.063	0.018 ~ 0.213
	Mostly satisfied	−1.721	0.491	12.272	0	0.179	0.068 ~ 0.469
	Neutral	−1.325	0.49	7.31	0.007	0.266	0.102 ~ 0.695
	Dissatisfied	−0.218	0.528	0.17	0.68	0.804	0.285 ~ 2.266
	Very dissatisfied	0	.	.	.	1.000	
Social support	0 individuals	0.572	0.339	2.85	0.091	1.772	0.912 ~ 3.438
	1–2 individuals	−0.01	0.261	0.001	0.971	0.990	0.594 ~ 1.65
	3–5 individuals	−0.323	0.336	0.92	0.337	0.724	0.375 ~ 1.399
	≥6 individuals.	0	.	.	.	1.000	
Activities of daily living (ADL)		−0.008	0.004	4.688	0.03	0.992	0.984 ~ 0.999
Years of caregiving experience		0.021	0.018	1.353	0.245	1.021	0.986 ~ 1.057
Weekly caregiving time		0.009	0.003	6.539	0.011	1.009	1.002 ~ 1.015

## 4 Discussion

This survey revealed that 47.9% of older adults with disabilities in Guangzhou exhibited severe limitations in activities of daily living (ADL). Comparatively, a study of community-dwelling older adults aged 70 and above in the United States reported a higher severe disability rate of 65.2% (10). The proportion of family caregivers experiencing moderate-to-severe burden in Guangzhou reached 42.8%, contrasting with findings from Shanghai, where a study of disabled older adults and their family caregivers documented a 24.95% prevalence of moderate-to-severe caregiver burden (11), indicating regional disparities in caregiver burden intensity. In this study, female family caregivers accounted for 62.3% of the total. Another survey on older adults individuals with disabilities conducted in communities in eastern China revealed a similar pattern, with female family caregivers comprising 66.7% of the sample (12).

Multivariate ordered logistic regression analysis identified longer weekly caregiving time as a significant risk factor. A Taiwan-based study of 2,439 caregivers demonstrated that those utilizing respite services (defined as structured programs providing caregivers with scheduled rest and recreational time) for over 14 days experienced substantially reduced caregiving burden compared to non-users (13). A study conducted in Japan targeting 82 family caregivers of older adults individuals with disabilities demonstrated that family caregiving services contributed to reducing the burden on caregivers (14). These findings underscore the necessity for governmental coordination of community resources, including neighborhood committees, volunteer networks, senior care service centers, and older adults associations, to establish comprehensive service systems. Implementation of daytime care services and structured respite programs could effectively reduce caregivers’ daily time commitment, enabling temporary relief from continuous caregiving responsibilities.

ADL capacity emerged as a protective factor against caregiver burden, aligning with findings from Nardi et al. (15). Strategic interventions should prioritize enhancing disabled older adults’ functional independence through rehabilitation training and assistive device implementation. Concurrent measures to prevent disability progression include regular health screenings for early detection of treatable conditions, coupled with encouragement of self-care activities within older adults’ physical capabilities to reduce caregiver dependency. The analysis further identified caregivers’ physical diseases as a significant risk factor, with caregivers’ mean age being 63.2 ± 12.0 years. Advanced age correlates with diminished physical stamina and increased health vulnerability, while caregiving itself exacerbates health deterioration (16, 17). A study investigating spousal caregiving for older adults individuals with disabilities found that regardless of caregivers’ willingness to provide care, negative experiences predominated in the caregiving process, particularly when caring for older adults spouses with particularly severe disabilities (18). This study found that 65.6% of the caregivers suffered from physical diseases. These results necessitate dual-focused health interventions: regular health monitoring, exercise promotion, sleep hygiene optimization, and nutritional guidance for caregivers, complemented by health education programs to enhance self-management competencies.

Higher life satisfaction (“very satisfied,” “mostly satisfied” or “neutral”) demonstrated statistically significant protective effects against caregiver burden, with threshold effects observed only at or above the “neutral” level (*p* < 0.05). Despite demonstrating a statistically significant association between life satisfaction and caregiver burden in our cross-sectional analysis, these findings cannot confirm causal directionality or reciprocal relationships. If causal precedence or reciprocal effects are substantiated, in-depth examination of the mediating pathways by which life satisfaction modulates caregiver burden becomes methodologically imperative.

Notably, 45.8% of caregivers reported no social interactions, and 6.3% lacked social support entirely. The effects of available social support networks and social frequency on family caregiver burden demonstrated no statistically significant difference. This suggests that caregivers’ social resources and engagement frequency may not effectively translate into protective factors for burden mitigation. Findings from a study involving 115 informal caregivers in Slovakia indicated an inverse correlation between caregiving burden and the degree of social support (19). A study utilizing data from the Ohsaki Cohort 2006 Study demonstrated that social support mitigates the risk of functional disability among caregivers (20). Another study indicates that participation in support groups can significantly alleviate stress levels among family caregivers (21).

Focusing on disabled older adults and their family caregivers within the megacity context of Guangzhou, this study provides direct evidence to inform differentiated health resource allocation policies for major urban centers. This study additionally incorporated long-term care insurance status as a variable. Notably, no statistically significant effect of long-term care insurance coverage on caregiver burden was observed. This underscores the imperative for developing more effective, multidimensional support strategies to alleviate caregiver burden, highlighting the persistent complexity of this public health challenge.

Intervention strategies may focus on multidimensional approaches to enhance caregiver well-being: establishing mutual support groups for experience-sharing among caregivers, enhancing community outreach programs through neighborhood committees and social workers to address practical challenges, and developing dedicated psychological counseling platforms for stress management. Policy initiatives should integrate these psychosocial components into municipal caregiver support frameworks while allocating resources for community-based implementation.

## 5 Limitations and prospects

Caregivers experiencing extreme exhaustion, severe time constraints, or resistance to participation had lower inclusion likelihood. Our sample likely underestimates the proportion of the most overwhelmed and time-pressured caregivers, potentially leading to underestimated burden levels. This study selected districts representing established downtown, new urban areas, and inner suburbs—three categories constituting an intra-urban core-periphery development continuum. However, outer suburban populations were not covered. It is suggested that comparative studies be carried out in the outer suburbs in the future.

Different types of illnesses experienced by family caregivers may exert differential effects on caregiver burden. Given the highly dispersed disease spectrum and the limited sample size for any single diagnostic category within the present study, stratifying caregivers by specific disease types would create numerous subgroups with insufficient statistical power for comparative analysis. It is recommended that future studies be designed to facilitate adequately powered comparisons across caregiver disease subgroups.

Filial piety culture may contribute to underestimation of caregiver burden. To more accurately capture the full panorama of caregiving burden within Chinese cultural contexts, future studies should incorporate validated scales specifically measuring filial stress and social desirability bias.

This study primarily investigated the intensity of caregiver burden, with particular focus on burden-exacerbating factors. However, integrating both positive and negative dimensions of the caregiving experience represents a critical future research direction. We recommend that subsequent investigations incorporate standardized scales measuring positive caregiving experiences alongside burden assessments, enabling comprehensive evaluation of caregiving.

## 6 Conclusion

Against the backdrop of population aging in China, caregiver burden among family members supporting disabled older adults warrants urgent attention. This study reveals a pervasive burden experienced by caregivers of the disabled old adults in Guangzhou. Empirical evidence confirms that caregiving time, caregivers’ physical diseases, disabled old adults’ activities of daily living (ADL) capacity, and caregivers’ life satisfaction constitute critical influence factors of burden severity. To address these challenges, targeted multi-level interventions are imperative. At the individual level, implementing ADL rehabilitation programs for care recipients is essential to enhance functional independence. Community-level strategies should prioritize establishing social support networks to improve caregivers’ psychosocial well-being. Institutionally, integrating caregiver health monitoring into the public health service system represents a vital policy direction.

While this study employed a methodologically sound design and implemented rigorous quality control measures—providing valuable reference evidence for alleviating family caregiver burden among disabled older adults—its cross-sectional nature precludes causal inference. Future cohort studies are warranted to validate the current findings.

## Data Availability

The datasets presented in this study can be found in online repositories. The names of the repository/repositories and accession number(s) can be found in the article/supplementary material.
